# Case Reflection of a Child With p.Phe312del/p.Phe508del Genotype Undetected on Newborn Screening and With No Clinical Features of Cystic Fibrosis Despite a Sweat Chloride Value in the Diagnostic Range

**DOI:** 10.7759/cureus.81151

**Published:** 2025-03-25

**Authors:** Jonathan Clarke, Nadeem Gadelsayed, Mohammed Elsammak, Jennifer Brady, Basil Elnazir

**Affiliations:** 1 Pediatrics, Children’s Health Ireland at Tallaght, Dublin, IRL; 2 Chemical Pathology, Children’s Health Ireland at Temple Street, Dublin, IRL; 3 Clinical Biochemistry, Children’s Health Ireland at Temple Street, Dublin, IRL; 4 Paediatric Respiratory Medicine, Children’s Health Ireland at Tallaght, Dublin, IRL

**Keywords:** becker’s muscular dystrophy, cystic fibrosis, newborn screening program, pediatric neurology, pediatric pulmonology

## Abstract

This clinical overview reflects on a case of a nine-month-old boy presenting with mild bronchiolitis and persistently elevated transaminases. A total creatine kinase (CK) was requested to assess for dystrophinopathies, which was significantly elevated at 3000 U/L on repeat samples. Molecular testing confirmed the diagnosis of Becker’s muscle dystrophy (BMD). During molecular testing, two cystic fibrosis (CF) mutations were incidentally detected, a p.Phe312del mutation and the classic CF-causing mutation p.Phe508del. Sweat chloride testing was repeatedly elevated in keeping with the diagnosis of CF. Despite the significantly elevated sweat chloride and molecular genetic profile showing heterozygosity for p.Phe508del and p.Phe312del mutations, the patient did not show any clinical manifestation of CF. During the newborn screening, immunoreactive trypsinogen (IRT) was 26 ng/mL, below the upper limit value used for screening (54 ng/mL) at that time. This case illustrates two important points: firstly, patients heterozygous for p.Phe312del and p.Phe508del mutations may not be detected during newborn screening and may not have clinical manifestations of cystic fibrosis despite having unequivocally elevated sweat chloride. Secondly, an unexplained elevation of transaminases should trigger creatine kinase testing to check for dystrophinopathies.

## Introduction

Cystic fibrosis (CF) is one of the most common autosomal recessive monogenic diseases that may present with significant morbidity and mortality [1]. Untreated patients have a much shorter life expectancy compared to unaffected subjects or patients who had earlier diagnosis and management. Without treatment, affected patients usually develop severe illness due to pulmonary and gastrointestinal complications of the disease and may succumb to death by the second or third decade of life [2]. However, early detection and treatment significantly improve the quality of life of affected patients and increase their life expectancy [3,4].

The incidence of CF is one in every 2570 live births in Ireland with an approximately one in 25 carrier rate [5]. The newborn screening program (NBS) was introduced in Ireland in July 2011. It involves measuring the immunoreactive trypsinogen (IRT) level on a dried bloodspot (DBS) sample taken during the first 72-120 hours of life. A cut-off value of 99th percentile of IRT is used. For any baby with an IRT value above the 99th percentile cut-off value, DBS is re-punched and repeated in triplicate. If the mean of the triplicate is higher than the cut-off value, the sample is sent for molecular confirmation of DNA causing mutations in a panel of the 38 most common genotypes in Ireland. If the latter reveals one or two pathogenic mutations, a patient is sent for sweat chloride testing as a gold standard for diagnosis.

The presentation of CF is dependent upon which organs are affected. Common presentations include chronic respiratory infections and malabsorption. Patients with atypical disease tend to present late in childhood or as adults. Herein, this paper reports on a case of CF that was undetected on NBS, where the patient presented with suspected myopathy at the age of nine months due to persistently elevated alanine aminotransferase (ALT).

## Case presentation

A nine-month-old Caucasian boy presented to the emergency care unit of Children’s Health Ireland at Tallaght in Dublin with symptoms suggestive of viral bronchiolitis. His parents reported no history of meconium ileus, prolonged neonatal jaundice, diarrhea, cough, or recurrent chest infections.

The patient’s clinical examination at the time was notable for mild respiratory distress with scattered crepitations and wheeze. He responded well to supportive bronchiolitis therapy with nebulized saline and low-flow supplemental oxygen.

Liver function tests revealed an elevated ALT level at 63 U/L (reference range: 10-45 U/L). The patient was not on any medications that might cause an increase in hepatic transaminases. This mild elevation of ALT was interpreted as a mild hepatic impairment secondary to the viral infection causing his bronchiolitis. He was discharged after the resolution of the acute infection. During follow-up visits, the transaminase level was repeated to ensure complete resolution; however, his ALT level was persistently elevated at 74 U/L.

The treating pediatrician suspected a muscle origin of the elevated transaminases and requested serum creatine kinase (CK), which was elevated at 800 U/L (upper limit: up to 160 U/L). The CK result was subsequently confirmed and repeated after two weeks, and the CK level was significantly elevated at 3000 U/L.

A neurology review was sought, and the review confirmed the normal acquisition of motor milestones, age-appropriate speech and language development, and normal clinical and neurological examination.

A dystrophinopathy was suspected, presenting in the pre-clinical phase owing to the finding of persistently unexplained raised transaminases. Parental consent was taken, and a request for genetic testing for dystrophinopathies was requested in the form of whole exome sequencing (WES), in an attempt to offer comprehensive screening given that at this time, the diagnosis was unclear.

The report came back positive with a comment of the following: “in-frame deletion consistent with Becker’s muscular dystrophy.” During molecular testing, two CF mutations were incidentally identified: “one pathogenic mutation and one possibly pathogenic variant in the cystic fibrosis transmembrane conductance regulator (*CFTR*) gene of your patient.” The variant c.935_937delTCT, p.Phe312del results in an in-frame deletion of three nucleotides and thus in the removal of one phenylalanine from three consecutive phenylalanines located in one of the transmembrane helices of the encoded CFTR protein. This variation was reported in a compound heterozygous state with other pathogenic CFTR variants (such as the known p.Phe508del), in several patients with CF.

The other detected variant, c.1521_1523delCTT, p.Phe508del, is the most frequently pathogenic variant (CF mutation class II) within Europeans with CF or CFTR-associated diseases. It results in the loss of one amino acid, which results in a misfolded protein that is not transported to the cellular membrane. Genetic analysis showed that both mutations were present in trans-allelic variants in our patient, and each parent carries one of the two variants detected. Sweat testing revealed a chloride result of 75 mmol/L, and a repeat sweat chloride testing after one month showed persistently elevated sweat chloride at 84 mmol/L (with levels equal to or above 60 mmol/L considered indicative of CF; intermediate levels between 40 and 59 mmol/L possibly indicating cystic fibrosis screen positive, inconclusive diagnosis {CFSPID}; and a level less than 40 mmol/L considered normal). An initial diagnosis of CF was made, in addition to Becker’s muscular dystrophy (BMD). Following this, further investigations are needed to rule out any possible false positive results, including a thyroid function test, a pituitary screen, and a detailed nutritional panel.

Upon receipt of the molecular result suggesting the diagnosis of CF, the NBS card of the baby was retrieved, and clinical details were examined thoroughly. The baby had a normal, uneventful delivery with no history of meconium ileus or blood transfusion. The IRT quality control on the day of the analysis of this infant’s DBS was reviewed and was within the expected range.

The IRT level on the screened dried blood spot was 28.75 ng/mL. There was no history or clinical details that might suggest a diagnosis of CF in this baby. No analytical or process-related reason was detected as a potential explanation for this case going undetected.

Currently, the patient is followed up at Children’s Health Ireland at Tallaght. He does not have any signs or symptoms of CF. Clinically, his chest examination is unremarkable with no wheeze or signs of infection. Chest X-ray showed normal aerated alveoli and clear costophrenic angles with no evidence of infection. The baby is well nourished with good developmental milestones. A sample for stool elastase was taken, and the result was normal at 500 µg/g (the normal reference range being >200 µg/g), making any CF-related malabsorption unlikely.

## Discussion

CF is one of the most common monogenic diseases that can affect life expectancy. It is due to a mutation in the cystic fibrosis transmembrane conductance regulator (CFTR) protein. An increasing number of babies that are screened positive using IRT are labelled with an inconclusive diagnosis (cystic fibrosis screen positive, inconclusive diagnosis) due to discordance between IRT level, genotype result, and the clinical presentation (phenotype). On the other hand, false negatives are serious incidents during newborn screening. Every effort should be made to reduce and explain any false negative results from newborn screening.

More than 2000 cystic fibrosis-related mutations have been detected in the *CFTR* gene [6]. However, fewer mutations have been labelled as disease-causing with clear genotypic-phenotypic correlations. Terlizzi et al. examined the genotype-phenotype correlation of 70 different mutations in CF patients, and they highlighted the importance of mutation type and its presence on the same or different allele of the *CF* gene [7].

The combination of two or more mutations in cis (not in trans) affects the phenotypic presentation of cystic fibrosis. They may be considered pathogenic mutations; however, they may have limited effect on the development of CF manifestation if they are present in cis not in trans. Parental genotype analysis revealed one variant in each parent. CF may have a different clinical presentation with a myriad of signs and symptoms depending on the affected organ [8,9]. The genotype-phenotype correlation is important with patients having compound homozygous for the common p.Phe508del mutation and other possibly pathogenic variants, especially those with p.Phe312del/p.Phe508del genotype.

Usually, elevated IRT during CF NBS is clear, and patients with two mutations for CF are picked up and diagnosed. Arrudi-Moreno et al. reported a positive correlation between IRT level and sweat chloride level in confirmed cases of CF [10]. However, in our case, IRT was significantly lower than the IRT cut-off value used to detect CF and did not correlate with the sweat chloride level. It is worth noting that despite newborn screening being an essential tool in public health, false negatives may still occur, meaning milder variants may be missed.

Atypical presentations of CF also depend on the type of pathogenic mutations detected during molecular testing. Patients with atypical disease may present later on during childhood with CF-related complications (e.g., frequent respiratory infections, malnutrition, malabsorption, or pancreatitis). Our patient presented with a persistently elevated level of serum ALT that may be due to the hepatic manifestation of CF. Of note, an abdominal ultrasound was noncontributory at this early stage.

Alternatively, an elevated ALT level found in the current case may be of muscle origin as a result of increased muscle breakdown and the release of ALT, aspartate aminotransferase (AST), and CK from the muscles of Becker’s muscular dystrophy patients. Nathwani et al. reported persistently high levels of ALT in patients with severe muscle injury with a persistently elevated AST/ALT ratio [11]. Furthermore, elevated transaminases are an indication to rule out dystrophinopathies [12].

Thus, the high level of ALT in the patient described above might be of hepatic or muscular origin. A careful follow-up of the current case is underway to check for the progression of any possible CF-related liver disease.

Raraigh et al. reported three adults with a similar mutation pattern to the above case, namely, the pathogenic classical p.Phe508del and the possibly pathogenic variant p.Phe508del [13]. Those patients did not show any manifestation of CF but had a consistently increased sweat chloride result. Moreover, they reviewed a database of 25 individuals with similar patterns of mutations; they showed consistent elevation of sweat chloride results with variable CF manifestations.

Another important observation from the California CF NBS registry indicated that individuals bearing p.Phe312del and either p.Phe508del or another pathogenic CF-causing variants or bearing p.Phe312del in the homozygosis state had consistently elevated sweat chloride but otherwise variable or absent symptoms of CF [13]. It was indicated that the p.Phe312del mutation allows the synthesis of mature, fully processed protein with preserved function, and this explains the variable or absent CF manifestations [12]. However, a substantial percentage of patients harboring the 18 p.Phe312del mutations and other bona fide CF-causing variants had exocrine pancreatic insufficiency and possibly decreased pulmonary function. The above patient did not have an exocrine function defect, with a stool elastase of 500 mg/g, or any pulmonary manifestations, as indicated by clinical examination and chest radiography.

However, careful management and follow-up (including spirometry, imaging, and clinical assessment) for patients harboring the p.Phe312del mutation are required to detect the clinical manifestations of CF if and when they emerge.

## Conclusions

This article reflects on a case of a child with an unusual p.Phe312del/p.Phe508del genotype that was not detected by CF NBS. This case was incidentally detected during screening for muscle panel to check for Becker’s muscular dystrophy-causing mutations. The patient was found to have a raised sweat chloride value, in the diagnostic range, but similar to previously published cases, the patient does not, as of yet, have clinical features of CF. There are several lessons to be learned from this case; firstly, it can be difficult to identify cases with certain genotypes, such as p.Phe312del/p.Phe508del, during NBS, unless there is a previous family history, as the IRT level may be totally normal. Secondly, isolated persistently increased ALT level may not necessarily be of hepatic origin and might be due to an increase in release from skeletal muscles, and it may warrant the exclusion of muscle pathology. This case is an important example of the need for the exclusion of dystrophinopathies in any unexplained elevation of transaminases. Thirdly, patients with p.Phe312del/p.Phe508del genotype may only present with an isolated increase in sweat chloride with no other clinical manifestations, as the p.Phe312del mutation allows the production of fully processed CFTR protein with preserved function.
